# Phenotypic and Genotypic Spectrum of Early-Onset Developmental and Epileptic Encephalopathies—Data from a Romanian Cohort

**DOI:** 10.3390/genes13071253

**Published:** 2022-07-15

**Authors:** Anca-Lelia Riza, Ioana Streață, Eugenia Roza, Magdalena Budișteanu, Catrinel Iliescu, Carmen Burloiu, Mihaela-Amelia Dobrescu, Stefania Dorobanțu, Adina Dragoș, Andra Grigorescu, Tiberiu Tătaru, Mihai Ioana, Raluca Teleanu

**Affiliations:** 1 Regional Centre of Medical Genetics Dolj, Emergency County Hospital Craiova, 200642 Craiova, Romania; anca.costache@umfcv.ro (A.-L.R.); amelia.dobrescu@umfcv.ro (M.-A.D.); stefania.crgm@gmail.com (S.D.); adina.crgm@gmail.com (A.D.); mihai.ioana@umfcv.ro (M.I.); 2 Laboratory of Human Genomics, University of Medicine and Pharmacy of Craiova, 200638 Craiova, Romania; andra.grigorescu97@gmail.com; 3 Department of Pediatric Neurology, Dr. “Victor Gomoiu” Clinical Children′s Hospital, 022102 Bucharest, Romania; eugenia.roza@umfcd.ro (E.R.); raluca.teleanu@umfcd.ro (R.T.); 4 Department of Pediatric Neurology, “Carol Davila” University of Medicine and Pharmacy, 050474 Bucharest, Romania; 5 Department of Pediatric Neurology, Prof. Dr. Alex. Obregia Clinical Hospital of Psychiatry, 041914 Bucharest, Romania; magda_efrim@yahoo.com (M.B.); iliescu_catrinel@yahoo.com (C.I.); carmenburloiu@gmail.com (C.B.); 6 Medical Genetics Laboratory, Victor Babes National Institute of Pathology, 050096 Bucharest, Romania; 7 Department of Genetics, Faculty of Medicine, Titu Maiorescu University, 031593 Bucharest, Romania; 8 Emergency County Hospital Targu Jiu, 210218 Târgu Jiu, Romania; tatarutiberiu@gmail.com

**Keywords:** developmental and epileptic encephalopathy, Dravet syndrome, generalized epilepsy with febrile seizures plus, NAV1.1 voltage-gated sodium channel

## Abstract

Early-onset developmental epileptic encephalopathy (DEE) refers to an age-specific, diverse group of epilepsy syndromes with electroclinical anomalies that are associated with severe cognitive, behavioral, and developmental impairments. Genetic DEEs have heterogeneous etiologies. This study includes 36 Romanian patients referred to the Regional Centre for Medical Genetics Dolj for genetic testing between 2017 and 2020. The patients had been admitted to and clinically evaluated at Doctor Victor Gomoiu Children’s Hospital and Prof. Dr. Alexandru Obregia Psychiatry Hospital in Bucharest. Panel testing was performed using the Illumina^®^ TruSight™ One “clinical exome” (4811 genes), and the analysis focused on the known genes reported in DEEs and clinical concordance. The overall diagnostic rate was 25% (9/36 cases). Seven cases were diagnosed with Dravet syndrome (likely pathogenic/pathogenic variants in *SCN1A*) and two with Genetic Epilepsy with Febrile Seizures Plus (*SCN1B*). For the diagnosed patients, seizure onset was <1 year, and the seizure type was generalized tonic-clonic. Four additional plausible variants of unknown significance in *SCN2A, SCN9A,* and *SLC2A1* correlated with the reported phenotype. Overall, we are reporting seven novel variants. Comprehensive clinical phenotyping is crucial for variant interpretation. Genetic assessment of patients with severe early-onset DEE can be a powerful diagnostic tool for clinicians, with implications for the management and counseling of the patients and their families.

## 1. Introduction

Epilepsy is a chronic disorder that poses a significant global burden due to its negative neurobiological, cognitive, psychological, and socio-economic impact on patients and their families. Epilepsy is estimated to have a combined incidence of ~195/100,000 live births in high-income countries and may have even higher incidence rates in low-income countries [1,2,3]. We could not identify reliable reports on the incidence of epilepsy in the Romanian population.

Early-onset epilepsy is usually associated with cognitive and behavioral changes, global developmental delay, pharmaco-resistant seizures, and high mortality [1,4,5,6,7]. To appropriately address the polymorphic etiology of the disease [8], the diagnosis must rely on the use of the latest gene testing, neuroimaging, metabolic, and immunological techniques to raise the successful diagnosis rate [9] from that which can be achieved by precise electro-clinical phenotyping alone [1,10].

Over the last two decades, genetic testing has become an essential tool for precise molecular diagnosis and prognosis, therapy choice, and appropriate counseling for family planning; the current case-series is yet another showcase. Rather numerous known Mendelian genes are targeted for diagnosis [11,12]. The underlying genetic complexity is generally not fully understood and mirrors the large phenotypic variability [13,14].

As a heterogeneous group of neurodevelopmental pathologies, epilepsy is more frequently discovered in the first year of life than in late childhood [10,15]. Age at onset is one of the main criteria used by the International League Against Epilepsy (ILAE) in the latest classification of epilepsy. Epilepsy syndromes are further classified based on the type of epilepsy into the following categories: focal; generalized; focal and/or generalized (e.g., Genetic Epilepsy with Febrile Seizures Plus, GEFS+); and developmental and epileptic encephalopathies (DEE) (e.g., Dravet syndrome, DS; etiology specific DEEs) [8]. Given this wide spectrum of epilepsy, one can easily see the importance of early recognition and the use of appropriate treatment modalities to improve an otherwise devastating outcome [16,17,18].

To our knowledge, data on genetic causes of early-onset childhood epilepsy in Romanians is scarce. Hence, we sought to extensively study a small group of patients who had a similar phenotype that closely overlaps the diagnosis criteria for DEEs. We present a case series of rare epilepsies in an ethnicity with few previously reported cases. Our diagnostic findings contribute to the significant strides being made in molecular diagnosis in managing genetic epilepsies.

## 2. Materials and Methods

### 2.1. Patients

This study includes 36 patients referred to the Regional Centre for Medical Genetics Dolj for genetic testing between 2017 and 2020. The presumptive diagnosis was genetic DEE.

The patients had been admitted and clinically evaluated at two tertiary clinics, Doctor Victor Gomoiu Children′s Hospital and Prof. Dr. Alexandru Obregia Psychiatry Hospital in Bucharest for the following:

(1) Onset of febrile or afebrile focal (usually hemiclonic), generalized tonic-clonic, myoclonic, or atypical/typical absence seizures before 36 months of age;
(2) Normal development before the onset of seizures;
(3) Intractable seizures requiring treatment with more than 2 antiepileptic drugs (AEDs);
(4) Exclusion of other epilepsy syndromes.

Patients had uneventful prenatal and neonatal history and normal neurological and developmental exams at onset. Over time, regression and neurological impairment comprising ataxia and pyramidal signs developed. Medical information, including seizure types, presence of febrile seizures, electroencephalography (EEG), and magnetic resonance imaging (MRI) reports, was obtained from referring clinicians.

### 2.2. Genetic Testing

Genetic testing was performed on DNA isolated with commercial kits from EDTA venous blood using next-generation sequencing (NGS). The “clinical exome” Illumina ^®^ TruSight ™ One was run on NextSeq550 IVD, MID-output to reach 100× mean coverage of targeted content. Library preparation and sequencing were performed according to the manufacturer’s instructions (Illumina, San Diego, CA, USA). The gene list is available at https://www.illumina.com/content/dam/illumina-marketing/documents/products/gene_lists/gene_list_trusight_one.zip (accessed on 1 July 2022). Paired-end 2 × 150 bp reads were mapped to the human genome reference sequence (GRCh37, iGenomes resource bundle) and pushed through the nf-core/sarek 2.7.1 pipeline. Mosaicism analysis was not performed for the probands or their progenitors. Validation for low coverage variants, de novo status, and/or segregation was offered with in-house capillary sequencing on a 3730xl DNA Analyzer (Life Technologies, Carlsbad, CA, USA).

### 2.3. Variant Interpretation

The targeted NGS gene panel includes genes associated with frequent and rare epilepsies among the ~4800 covered genes. The germline variants identified were annotated using ENSEMBL variant effect predictor (VEP) [19], with several plugins for predictive scores [20]; online aggregate databases such as OMIM, ClinVar, and Varsome were also consulted [21,22,23]. Annotated and inheritance information, where available, were used for ACMG compliant variant classification [24]. Variants with a depth of over 20× were considered for diagnosis. Additionally, the coverage of several genes was manually investigated. If necessary, capillary sequencing was used to get full coverage of exons in genes of interest.

We considered positive results to be the presence of pathogenic/likely pathogenic variants in genes that explained the testing indication: (1) in a heterozygous state for dominant conditions; (2) a homozygous or compound heterozygous for recessive conditions; (3) and a hemizygous variant in an X-linked recessive gene in males. We are also discussing variants of unknown significance that may be plausible in the clinical context.

## 3. Results

### 3.1. Phenotypic Description

Our study comprised 36 patients, 23 males and 13 females, ranging from 1 to 15 years old at the time of most recent clinical evaluation. For all cases, seizure onset was between 6 and 36 months.

Twelve patients had compelling clinical evidence of DEE with developmental delay reported after epilepsy onset; out of these, 4 reported isolated speech delay after seizure onset.

A positive family history of epilepsy was reported in 2 probands: (1) a first-degree family member with DS; and (2) a second-degree family member with epilepsy of unknown etiology. A family history of febrile seizures was reported in 4 additional probands.

Seizure onset occurred at an average age of 12.7 months, followed by generalized tonic-clonic seizures, focal seizures (tonic or clonic), and epileptic myoclonus, and the majority of the patients had atypical/typical absence seizures. Common additional seizure types included focal tonic or focal clonic seizures grouped as other focal seizures. All patients with documented episodes of status epilepticus experienced prolonged generalized or hemiconvulsive seizures. Mainly generalized seizures were reported.

The referring clinician reported intellectual disability and behavioral problems in 6 of 36 patients. Deep phenotyping with formal cognitive testing was available (WPPSI/Wechsler Intelligence Scale for Children) and showed 2 patients with moderate to severe ID (intelligence quotient (IQ) < 70), 2 with mild ID (IQ = 70–85), and 2 as normal (IQ > 85). Thirty patients had missing information about deep phenotyping with formal cognitive testing.

The main phenotypic and genotypic findings for our study group are summarized in Table 1. As shown below, the phenotypic configuration led us to segregate patients with variants in the *SCN1A* genes from patients with variants in other genes.

### 3.2. Genetic Findings

The variants identified in this Romanian cohort are described in Table 2. We are reporting variant description, ClinVar data, and ACMG classification criteria met based on current knowledge, and their interpretation in the context of the suggested disorder.

All identified variants were heterozygous. An autosomal dominant inheritance pattern has been described in associated neurological disorders that overlapped with the clinical data. Despite good coverage, the identified genetic variants are rare, and either could not be found in variation databases or had extremely low frequencies. Segregation data and *de novo* status are not included due to a lack of family compliance at the date the article was published.

We were able to identify promising variants in genes that correlated with the reported phenotype in 13/36 patients (36.11%). If we restrict to pathogenic/likely pathogenic ACMG scores, the diagnosis success rate becomes 9/36 (25%). Seven of the variants have not been previously reported, to our knowledge.

As ethnicity-based comparisons are scarce in the literature, we summarized the clinical presentation of *SCN1A* mutations in DS in Supplementary Table S1. We are presenting the clinical features described in European studies published over the last 11 years that we identified. The phenotype described in our cohort has similar traits to those described in Table S1. Unfortunately, the larger studies, in particular, did not include clinical information.

## 4. Discussion

### 4.1. Phenotype in DEEs

Epileptic syndromes are defined according to specific electro-clinical phenotypes: particular seizure types, the typical age at onset, EEG, and brain imaging features [11]. In early-onset childhood epilepsy, it is particularly challenging to establish a clinical diagnosis during the first examinations, especially when few of the epileptic syndrome criteria are met. Despite these diagnostic pitfalls, early recognition of “possible epilepsy syndromes” is a tremendous asset as it is a difficult endeavor.

For the clinical presentation, the overlap with the phenotype described in the literature is striking, especially for the DS subgroup. Our study mostly concurs with the clinical features that other studies report [33] in >85% DS cases: seizure onset during the first year of life, multiple seizure types, fever as a trigger for seizures, abnormal EEG, normal development initially and later developmental delay, and drug resistance to multiple antiepileptics. More recent reports on the European population, presented in Supplementary Table S1, seem to align with the common findings above. Of note, age of onset in the primary year remains a defining characteristic for DS in our case series too.

Despite the phenotypic polymorphism and the small number of subjects included in our study, we describe some clinical consistency, depending on the gene involved [12]. Our case series describes strikingly similar patient characteristics between the subgroups with *SCN1A* variants identified, with variants in other DEE reported genes, or without a genetic diagnosis at this point, which is yet further evidence that defining the electro-clinical phenotype alone cannot elucidate the etiological diagnosis.

### 4.2. Genetic Findings in Our Study and Their Clinical Correlates

Despite the highly similar electro-clinical phenotypes of the enrolled patients, the results obtained through NGS-based tests revealed possible variants in the *SCN1A* gene and four other epilepsy-related genes (*SCN1B, SCN2A, SCN9A,* and *SCL2A1*). Genes such as these encode sodium channels and are associated with many cases of drug-resistant genetic epilepsies with onset in infancy and childhood [34,35].

According to ACMG, nine candidate variants with clinical concordance were assessed to be pathogenic/likely pathogenic. Four additional variants were initially assessed as having uncertain significance and were considered possibly causative in the clinical context.

### 4.3. SCN1A Cases

Perhaps the most studied representative of DEEs is Dravet syndrome (DS)–DEE-6A, formerly known as Severe Myoclonic Epilepsy of Infancy (SMEI). This is a rare and severe genetic disorder of infancy-onset febrile epilepsy.

For most of the patients, we were able to identify *SCN1A* variants, with DS being the most frequent pathology in our group. DS represents one of the most documented genetic disorders of infancy-onset febrile epilepsy, which is considered to be a rare and serious condition. DS is noted for its intractable seizure polymorphism, the considerable risk of status epilepticus it presents, and its inevitable progression towards neurodevelopmental deficits and impaired gait later in life [8,30].

The cause of DS is widely accepted to be *de novo* [30,36,37] mutations involving the SCN1A gene [30,38]. The gene encodes for the α subunit of a voltage-gated neuronal sodium channel (NaV1.1), and its mutations lead to varying degrees of channel loss of function [39]. DS follows a model of haploinsufficiency for its pathogenesis [33]. Unfortunately, the lack of information on the de novo status is a limitation of our study. Should families be willing, we hope to perform extended targeted testing.

In DS there is a higher rate of truncated proteins, which our study also found. This generated the supposition of a direct correlation between the severity of the mutation, the scope of the NaV1.1 channel loss-of-function, and the severity of the phenotype. In contrast, some authors show that, occasionally, the same variant produces a wide phenotype variety in either DS or GEFS+ patients [40,41]. Cases where the same mutation has led to both GEFS+ and DS have been reported, even among members of the same family [26,42,43]. A possible explanation may be the position of the mutation in relation to the pore-forming region of the channel, as those in this region are more likely to be specific to DS, and those outside the region, to GEFS+ [44]. For all seven patients reported in our study, a diagnosis of DS stands given the clinical correlates.

An unaddressed issue in the current study remains the case of mosaic *SCN1A* variants that may explain the variable expression of the phenotype [26,45,46]. Given the compelling literature, we believe this needs to be part of the genetic evaluation. We plan to implement an NGS analysis pipeline and laboratory validation (MIPs and/or digital PCR) so that further testing may shed light on this aspect.

As is often the case with many genes, the *SCN1A* gene can be responsible for multiple phenotypes: DS, GEFS+, and DEE-6B. The definite cause of DS or DS-like and its delineation from GEFS+ are proven to be far more complex phenomena, as other genes such as *PCDH19, GABRG2,* and *GABRA1* may also play a role [47,48,49,50].

### 4.4. Other Cases

Heterozygous potentially pathogenic variants of *SCN1B* have been reported in patients with DS phenotype or genetic epilepsy with febrile seizure plus, showing variable penetrance or absence epilepsy [51]. The *SCN1B* variant reported in our study was identified in two of the patients. Clinical presentation and plausible inheritance point toward a diagnosis of GEFS+ for this autosomal-dominant heterozygous variant.

The large spectrum of *SCN2A*-related epilepsy includes epilepsy with a comparatively favorable prognosis and epileptic encephalopathy [51,52,53]. The clinical presentation of *SCN2A*-bearing individuals includes early-onset focal cluster seizures [51,52,54]. In our study, the patient identified with a missense VUS variant in the *SCN2A* gene presented atypical absence of seizures at the age of 24 months.

There are studies that report *SCN9A* variants to be associated with dominantly inherited febrile seizures or to play a role in the modulation of phenotype in DS patients [55]. Thus, it is possible that variants of unknown significance affecting *SCN9A* act as a modifier in the presence of *SCN1A* variants [31,55]. Our study could not identify additional *SCN1A* variants or other pathogenic or likely pathogenic variants for the two cases where missense VUS variants of *SCN9*A were reported.

*SLC2A1* is the only gene associated with GLUT1 deficiency syndrome—DEE, a rare and potentially treatable condition inherited in an autosomal-dominant manner with complete penetrance [56,57,58]. The early recognition of this DEE is essential to correctly manage the cases, and the avoidance of trigger events such as prolonged fasting and intense physical efforts are recommended in affected patients. However, functional studies have shown that the identified variant mildly impacts the glucose transporter function. Available genetic data do not support strong segregation with the disease [32], which explains the VUS classification. The *GLUT1* reported case in our study did not manifest the classical phenotype with developmental and epileptic encephalopathy. He showed only a few typical GLUT1-deficiency syndrome features, such as seizures and EEG alterations.

### 4.5. Diagnosis Rate and Choice of a Genetic Test in Early-Onset Epilepsy

The overall diagnosis rate in our study was 25%, which falls within the reported range estimated between 13 and 28% [59,60]. The reported diagnosis rate varies widely due to different inclusion criteria and testing options: targeted gene panels, clinical exome-sequencing (CES), whole-exome sequencing (WES), and less frequent whole-genome sequencing (WGS), which in some cases were followed by capillary sequencing. The chosen approach may greatly influence diagnostic accuracy and success rates.

Some authors suggest that the conventional way of initially sequencing the strongest candidate gene should be reconsidered in cases of suggestive clinical manifestations [36]. A variety of papers used targeted gene panels, and NGS has been recommended as an essential second step for *SCN1A*-negative patients following Sanger sequencing, as technical errors may occur with the classical technique [61,62,63]. The cost-efficiency of this approach needs to be proven. Nevertheless, in the case of the *SCN1A* gene and not only, NGS has also played a critical role in identifying relevant novel mutations [49,63,64,65,66].

In some studies, gene panels have a diagnostic yield that is comparable with WES [67], whereas in other studies, WES has a superior yield [55,68,69]. Clinical exome sequencing is also widely used. Generally, the analysis uses a “gene panel” approach that filters data on prior knowledge. Including more genes can be attractive in that it can potentially uncover novel gene–disease associations [63,70].

In our study, most of the identified variants were rare, novel ones, involving *SCN1A* in 7/9 cases. We believe though that the similar clinical presentation in our patients warrants an extensive search for genetic etiology. Our literature-based rationale for choosing a “clinical exome” as a diagnostic tool was based on several considerations: (1) it includes a high number of known candidate genes for EEs, including DS [36,64,65], (2) reported success rates are comparable to the ones mentioned above [71], up to 50% [72], and (3) it allows for the discovery of novel gene–disease correlations.

The downside of WES is that it does not always guarantee a full coverage of every exon [59], and deletion/duplication would require additional testing (e.g., MLPA). In our case, for the genes with plausible hits, we manually looked for coverage in all evaluated patients. Our study did not evaluate deletions/duplications.

As it stands, monogenic variants are a substantial part of DEE etiology. This can paint a skewed picture of the underlying genetic architecture of this condition, leaving no room for oligogenic or modifier variants, for instance [73]. This warrants more extensive sequencing and periodic revisiting of the generated data [60]. WES may not currently be a viable first-line test in many settings, but for these reasons, it would be beneficial to consider its use early in the work-up of epilepsy [59].

The unprecedented rate of NGS use leads to the considerable number of variant discoveries of unreliable significance in the clinical context [61]. We also chose to only include several promising VUS in our reporting and discussions [74]. As this study also shows, tackling these particularities of NGS analysis adds another layer of difficulty to genetic diagnosis.

In the absence of structured guidelines, genetic testing relies heavily on the clinical judgment and, in countries like Romania, on the availability and affordability of tests. Algorithms to set geneticists, genetic counselors, and neurologists on the same page are needed, as are viable protocols for identifying genetic causes and guiding the clinician on the choice of test [11,59,69].

Revealing the molecular cause is essential for diagnosis, treatment strategies, family genetic counseling, and public health strategies. To our knowledge, this study is among the first to contribute to these efforts in Romania.

## 5. Conclusions

Comprehensive clinical phenotyping is crucial for interpreting results. Genetic postnatal assessment of patients with severe early-onset epileptic encephalopathy and developmental delay can be a powerful diagnostic tool for clinicians and has implications for the management and counseling of patients and their families.

NGS-based diagnostic methods have the potential to provide an early diagnosis and drive therapeutic management to include empirically proven therapy or trials of new or targeted molecules. There are no practice guidelines regarding the best approaches to clinical genetic testing of individuals with epilepsy. This study is among the first to report early-onset DEE phenotype and genotype correlations in a Romanian population.

## Figures and Tables

**Table 1:** Neurological features and genetic findings of the tested patients: the study group, then segregated by genetic findings, with variants in the *SCN1A* gene and with variants in other genes.

Clinical Phenotype	Study Group n = 36	Phenotype of Our Patients Diagnosed with Likely Pathogenic/Pathogenic Variants n = 9	Phenotype of Our Patients Undiagnosed or Diagnosed with VUS Variants n = 27
**Sex**	23 M/13 F	5 F/4 M	8 F/19 M
**Seizure onset age (months)**	13.9 (M)/8.7 (F)	7 mo	14 mo
**Seizure semiology**	Generalized tonic-clonic seizures: 23/36 (63.89%) Generalized tonic seizures: 4/36 (11.11%) Generalized clonic seizures: 2/36 (5.56%) Myoclonic seizures: 3/36 (8.33%) Atypical absence seizures: 3/36 (8.33%) Typical absence seizures: 1/36 (2.78%)	Generalized tonic-clonic seizures: 4/9 (44.44%) Generalized tonic seizures: 1/9 (11.11%) Generalized clonic seizures: 1/9 (11.11%) Myoclonic seizures: 1/9 (11.11%) Atypical absence seizures: 1/9 (11.11%) Typical absence seizures: 1/9 (11.11%)	Generalized tonic-clonic seizures: 19/27 (70.37%) Generalized tonic seizures: 3/27 (11.11%) Generalized clonic seizures: 1/27 (3.70%) Myoclonic seizures: 2/27 (7.40%) Atypical absence seizures: 2/27 (7.40%)
Electrophysiology-interictal EEG *	Normal: 25/34 (73.53%) EEG with epileptiform discharges: 9/34 (26.47%)	Normal: 7/9 (77.78%) EEG with epileptiform discharges: 2/9 (22.22%)	Normal: 18/27 (66.67%) EEG with epileptiform discharges: 7/27 (25.93%) 2 NA (7.40%)
**Seizure-inducing factors**	Fever/infections/vaccines: 34/36 (94.44%)	Fever/infections/vaccines: 9/9 (100%)	Fever/infections/vaccines: 25/27 (92.59%) 2/27 NA (7.40%)
**Status epilepticus at onset/in evolution**	12/36 (33.33%)	4/9 (44.44%)	8/27 (29.63%)
**Intellectual disability/global developmental delay**	4/36 (11.11%)	3/9 (33.33%)	1/27 (3.40%)
**Speech delay/no speech**	8/36 (22.22%)	3/9 (33.33%)	5/27 (18.52%)
**Behavioral issues**	4/36 (11.11%)	2/9(22.22%)	2/6 (33.34%)
Neuroimaging findings *	Normal brain MRI: 33/35 (94.29%) Brain atrophy: 1/35(2.86%) Demyelinating lesion in the left frontal area: 1/35(2.86%)	Normal brain MRI: 8/9 (88.89%) Brain atrophy: 1/9 (11.11%)	Normal brain MRI: 26/27 (96.60%) Demyelinating lesion in the left frontal area: 1/27 (3.40%)
**Other health issues**	Motor delay: 4/36 (11.11%) Macrocephaly: 2/36 (5.56%) Microcephaly: 1/36 (2.78%)	Motor delay: 3/9 (33.33%) Microcephaly: 1/9 (11.11%)	Motor delay: 1/27 (3.40%) Macrocephaly: 2/27 (7.40%)
**Prenatal history**	Normal pregnancy history: 33/36 (91.67%)	Normal pregnancy history: 9/9 (100%)	Normal pregnancy history: 24/27 (88.88%) 3/27 NA (11.12%)
**Familial history of seizures/epilepsy**	6/36 (16.67%)	1/9 (11.11%)	5/27 (18.52%)
**Parental consanguinity**	No	-	-
**Genetic findings**	*SCN1A*: 7/36 (19.44%) *SCN1B*: 2/36 (5.56%) *SCN2A*: 1/36 (2.78%) *SCN9A*: 2/36 (5.56%) *SLC2A1* (*GLUT1*): 1/36 (2.78%)	*SCN1A*: 7/9; *SCN1B:* 2/9	*SCN2A:* 1/27; *SCN9A:* 2/27; *SLC2A1* (*GLUT1*): 1/27

* missing cases. M—males; F—females.

**Table 2:** Identified variants—MANE transcript, HGVS nomenclature, variant type, ClinVar information, ACMG score, and relevant literature on the variant. We are showing our patients the matching diagnosis given the clinical presentation.

Gene Variant	Variant Type	NCBI ClinVar	ACMG Score	Relevant Literature	Associated Phenotype
*SCN1A* NM_001165963.4: c.1285C > T p.Gln429Ter	Null variant (nonsense), exon 12 of 29, position 115 of 207 (coding, NMD)	Pathogenic, 1 star (criteria provided, 1 submission), associated with developmental and epileptic encephalopathy, early infantile epileptic encephalopathy, early infantile epileptic encephalopathy with suppression bursts, and Ohtahara syndrome	Pathogenic (PVS1, PM2, PP5)	-	Patient 7 DS
*SCN1A* NM_001165963.4: c.2134C > T p.Arg712Ter	Null variant (nonsense), exon 15 of 29, position 91 of 133 (coding, NMD)	Pathogenic, 2 stars (criteria provided, multiple submitters, no conflicts) associated with severe myoclonic epilepsy in infancy, early infantile epileptic encephalopathy with suppression bursts, generalized epilepsy with febrile seizures plus, type 2, autosomal dominant epilepsy, epileptic encephalopathy, early infantile, 1	Pathogenic (PVS1, PP5, PS3, PM2)	[25,26]	Patient 26 DS
*SCN1A* NM_001165963.4: c.2208del p.Cys737ValfsTer10	Null variant (frameshift), exon 16 of 29, position 32 of 239 (coding, NMD)	Not reported	Pathogenic (PVS1, PM2)	-	Patient 12 DS
*SCN1A* NM_001165963.4: c.2958_2959del p.Phe987SerfsTer9	Frameshift, exon 19 of 29, position 12–13 of 483 (coding, NMD)	Likely pathogenic (criteria provided, single submitter) associated with severe myoclonic epilepsy in infancy, generalized epilepsy with febrile seizures plus type 2	Likely pathogenic (PVS1, PM2, PP5)	-	Patient 13 DS
*SCN1A* NM_001165963.4: c.3718del p.Ile1240TyrfsTer30	Frameshift, exon 22 of 29, position 13 of 174 (coding, NMD)	Not reported	Likely pathogenic (PVS1, PM2)	-	Patient 19 DS
*SCN1A* NM_001165963.4: c.5515C > G p.Leu1839Val	Missense, exon 29 of 29, position 663 of 6610 (coding)	Pathogenic, 1 star (criteria provided, single submitters) associated with severe myoclonic epilepsy in infancy	Pathogenic (PM1, PM5, PM2, PP2, PP3, PP5)	[27]	Patient 14 DS
*SCN1A* NM_001165963.4: c.5536_5539del p.Lys1846SerfsTer11	Null variant (frameshift), exon 29 of 29, position 684–687 of 6610 (coding, NMD)	Pathogenic, 2 stars (criteria provided, multiple submitters, no conflicts) associated with severe myoclonic epilepsy in infancy, early infantile epileptic encephalopathy with suppression bursts, generalized epilepsy with febrile seizures plus, type 2	Pathogenic (PVS1, PM2, PP5)	[27,28,29,30]	Patient 8 DS
SCN1B* NM_001037.5: c.655T > C p.Ter219Glnext*214	Stop loss, exon 5 of 6, position 65 of 72 (splicing, coding)	Not reported	Likely pathogenic (PM4, PM2, BP4)	-	Patient 5, 28 GEFS+
*SCN2A* NM_001040143.2: c.5211C > A p.His1737Gln	Missense, exon 28 of 28, position 389 of 3676 (coding)	Not reported	Uncertain significance (PM2, PP3)	-	Patient 16 DEE 11
*SCN9A* NM_001365536.1: c.1675G > A p.Gly559Ser	Missense, exon 12 of 27, position 73 of 339 (coding)	Uncertain significance, 2 stars (criteria provided, multiple submitters, no conflicts) associated with generalized epilepsy with febrile seizures plus, type 7	Uncertain significance (PM2, PP3)	[31]	Patient 10 GEFS+
*SCN9A* NM_001365536.1: c.5693G > A p.Arg1898His	Missense, exon 27 of 27, position 919 of 4680 (coding)	Not reported	Uncertain significance (PM2, PP3)	-	Patient 36 GEFS+
*SLC2A1* (*GLUT1*) NM_006516.4: c.179C > T p.Thr60Met	Missense, exon 3 of 10, position 65 of 161 (coding)	Uncertain significance, 2 stars (criteria provided, multiple submitters, no conflicts) associated with GLUT1 deficiency syndrome 1, autosomal recessive	Uncertain significance (PM2, PP2)	[32]	Patient 34 GLUT1 deficiency syndrome-DEE

* variant identified in two apparently unrelated individuals. MANE—Matched Annotation from NCBI and EMBL-EBI; ACMG—American College of Medical Genetics; *SCN1A*—sodium voltage-gated channel α subunit 1; *SCN1B*—sodium voltage-gated channel β subunit 1; *SCN2A—*sodium voltage-gated channel α subunit 2; *SCN9A*—sodium voltage-gated channel α subunit 9; *SLC2A1* (*GLUT1*)—solute carrier family 2 member 1, glucose transporter 1.

## Data Availability

Not applicable.

## Supplementary Materials

The following supporting information can be downloaded at: https://www.mdpi.com/article/10.3390/genes13071253/s1, Table S1: Clinical phenotype as described in studies on European populations with SNC1A mutations diagnosed with Dravet syndrome (DS).
