# Supplementary material for: Phenotypic and Genotypic Spectrum of Early-Onset Developmental and Epileptic Encephalopathies—Data from a Romanian Cohort
Source: Genes (Basel). 2022 Jul 15;13(7):1253. doi: 10.3390/genes13071253 (PMC9322987; doi:10.3390/genes13071253)
Supplement: Supplementary file 1 [file genes-13-01253-s001.zip › genes-1740262-supplementary.pdf]

Table S1. Clinical phenotype of Dravet Syndrome (DS) cases, as described in studies on European populations with SNC1A mutations

**CLINICAL PHENOTYPE / STUDY**	Cetica, V. et al. (2017). [1]	Brunklaus, A et al. (2012). [2]	Nabbout, R. et al. (2013). [3]	Ragona, F. et al. (2010). [4]	Till, Á. et al. (2020). [5]	Peycheva, V. et al. (2020). [6]	Nabbout, R. et al. (2020). [7]	Johannesen, K. M. et al. (2020). [8]	Symon DS, J. D. et al. (2019). [9]	Bertok, S. et al. (2017). [10]	Stranneheim, H. et al. (2021). [11]
**Country**	Italy	UK	France	Italy	Hungary	Bulgaria	France	Denmark	UK	Slovenia	Sweden
**Study group (n)**	200 SCN1A+, 97 DS	241 SCN1A+, DS	67 DS, 58 SCN1A+	37 DS, 31 SCN1A+	63	60/ 22 SCN1A+, 10 DS	91 DS, 71 SCN1A+	200 (various pathologies), 17 DS	343 (263 various epilepsy syndromes), 11 DS	216	4437 (3219 patients, 1218 relatives)
**Males**	109/200	135/241	39/67	16/37	-	28/60	46/91	-	-	91/216	-
**Seizure onset age (months)**	10.8	6.0 (+/- 1.5)	4	5.7 (+/- 2.1)	6.0 *	8.81 *	5.12	10 **	6.5 *	30.72	-
**Seizure semiology**											
Generalized tonic-clonic	-	-	59 (88.1%)	37/37 (100%)	-	11/11 (100%)	87/87 (100%)	-	-	-	-
Generalized clonic seizures	-	-	45 (67.2%)	-	-	-	-	-	-	-	-
Generalized tonico-clonic and clonic seizures	142/168 (84.52%)	216/231 (93.5%)	-	-	-	-	-	-	-	-	-
Myoclonic seizures	5/168 (2.98%)	161/232 (69.4%)	35 (52.2%)	27/37 (72.97%)	-	2/11 (18.18%)	87/87 (100%)	-	-	-	-
Atypical absence seizures	-	112/218 (51.38%)	-	25/37 (67.57%)	-	-	87/87 (100%)	-	-	-	-
Typical absence seizures	-	-	-	-	-	-	-	-	-	-	-

(typical+atypical/ could be either)Absence seizures	1/168 (0.6%)	-	37 (55.2%)	-	-	1/11 (9.09%)	-	-	-	-	-
Status epilepticus at onset /in evolution	-	188/235 (80%)	52 (77.6%)	33/37 (89.19%)	7/15 (46.67%)	8/11 (72.73%)	-	-	5/14 (35.71%)	-	-
**Electrophysiology - interictal EEG**	38/88 (43.18%)		-	37/37 (100%)	-	-	-	-	-	-	-
**Seizure-inducing factors**											
Fever	134/167 (80.24%)	134/230 (58.26%)	58 (86.6%)	37/37 (100%)	17/17 (100%)	8/11 (72.73%)	-	-	7/14 (50%)	-	-
Infections	-	-	-	-	-	-	-	-	-	-	-
Vaccines	-	17/230 (7.39%)	-	-	-	-	-	-	-	-	-
**Intellectual disability**	-	-	63/67 (94.03%)	32/37 (86.48%)	6/10 (60%)	7/11 (63.64%)	80.5%	-	-	-	-
**Global developmental delay**	-	-	66/66 (100%)	-	4/16 (25%)	-	-	-	13/14 (92.85%)	-	-
**Speech delay / no speech**	-	-	31/58 (53.45%)		-	2/11 (18.19%)	54/91 (59.34%)	-	-	-	-
**Behavioral issues**	-	98/213 (46.01%)	-	21/37 (56,75%)	-	2/11 (18.19%)	NA	-	-	-	-
**Motor delay**	-	77/214 (35.98%)	66/66 (100%)	37/37 (100%)	1/14 (7,14%)	5/11 (45.45%)	57/91 (62.64%)	-	-	-	-
**Abnormal brain MRI**	-	22/200 (11%)	-	13/36	-	1/11 (9.09%)	-	-	-	-	-
**Normal Brain MRI**	-	0.89	-	23/36 (64%)	-	-	-	-	-	-	-

**Brain atrophy**	-	7/22 (31.82%)	-	5/36 (13,88%)	-	-	-	-	-	-	-
**Familial history of seizures / epilepsy**	111/199 (55.78%)	65/223 (29.15%)	25/67 (37.31%)	16/35 (46%)	-	4/11 (36.36%)	-	-	-	-	-
**Genetic diagnosis method**	direct sequencing; next gen amplicon sequencing, confirmed by Sanger sequencing; mutation negative samples - MLPA	standard sequencing; mutation negative samples - MLPA	direct sequencing; mutation negative samples - MLPA	DHPLC, MLPA	direct sequencing, MLPA	Sanger sequencing, segregation analysis	NA	NGS - various gene panels	custom-designed 104-gene epilepsy panel; Sanger sequencing and MLPA of 10 genes	fluorescence-based competitive allele-specific (KASPar) assay	NGS - clinical WGS

* for cases with SCN1A mutations
* for genetically diagnosed cases

References

1. Cetica, V., et al., *Clinical and genetic factors predicting Dravet syndrome in infants with SCN1A mutations.* Neurology, 2017. **88**(11): p. 1037-1044.
2. Brunklaus, A., et al., *Prognostic, clinical and demographic features in SCN1A mutation-positive Dravet syndrome.* Brain, 2012. **135**(8): p. 2329-2336.
3. Nabbout, R., et al., *Encephalopathy in children with Dravet syndrome is not a pure consequence of epilepsy.* Orphanet journal of rare diseases, 2013. **8**(1): p. 1-8.
4. Ragona, F., et al., *Dravet syndrome: early clinical manifestations and cognitive outcome in 37 Italian patients.* Brain and Development, 2010. **32**(1): p. 71-77.
5. Till, Á., et al., *Mutation spectrum of the SCN1A gene in a Hungarian population with epilepsy.* Seizure, 2020. **74**: p. 8-13.
6. Peycheva, V., et al., *SCN1A mutation spectrum in a cohort of Bulgarian patients with GEFS+ phenotype.* Turkish Journal of Pediatrics, 2020. **62**(5).
7. Nabbout, R., et al., *Impact of childhood Dravet syndrome on care givers of patients with DS, a major impact on mothers.* Epilepsy & Behavior, 2020. **108**: p. 107094.
8. Johannesen, K.M., et al., *Utility of genetic testing for therapeutic decision ‐ making in adults with epilepsy.* Epilepsia, 2020. **61**(6): p. 1234-1239.
9. Symon DS, J.D., et al., *Incidence and phenotypes of childhood-onset genetic epilepsies: a prospective population-based national cohort.* Brain, 2019. **142**(8): p. 2303-2318.
10. Bertok, S., et al., *The association of SCN1A p. Thr1067Ala polymorphism with epilepsy risk and the response to antiepileptic drugs in Slovenian children and adolescents with epilepsy.* Seizure, 2017. **51**: p. 9-13.
11. Stranneheim, H., et al., *Integration of whole genome sequencing into a healthcare setting: high diagnostic rates across multiple clinical entities in 3219 rare disease patients.* Genome medicine, 2021. **13**(1): p. 1-15.
